# Mesenchymal stromal cells integrate and form longitudinally-aligned layers when delivered to injured spinal cord via a novel fibrin scaffold

**DOI:** 10.1016/j.neulet.2014.03.023

**Published:** 2014-05-21

**Authors:** Alex J.T. Hyatt, Difei Wang, Christian van Oterendorp, James W. Fawcett, Keith R. Martin

**Affiliations:** a John van Geest Centre for Brain Repair, University of Cambridge, Robinson Way, Cambridge CB2 0PY, United Kingdom; b University Eye Hospital, Freiburg, Germany; c Cambridge NIHR Biomedical Research Centre, UK; d Wellcome Trust-MRC Cambridge Stem Cell Institute, UK

**Keywords:** Spinal cord injury, Mesenchymal stromal cells, Cell scaffold, Fibrin, MSCs, mesenchymal stromal cells, EDC, N-(3-dimethylaminopropyl)-N-ethyl-carbodiimide hydrochloride, NHS, N-hydroxysuccinimide

## Abstract

Mesenchymal stromal cells (MSCs) have been shown to promote healing and regeneration in a number of CNS injury models and therefore there is much interest in the clinical use of these cells. For spinal cord injuries, a standard delivery method for MSCs is intraspinal injection, but this can result in additional injury and provides little control over how the cells integrate into the tissue. The present study examines the use of a novel fibrin scaffold as a new method of delivering MSCs to injured spinal cord. Use of the fibrin scaffold resulted in the formation of longitudinally-aligned layers of MSCs growing over the spinal cord lesion site. Host neurites were able to migrate into this MSC architecture and grow longitudinally. The length of the MSC bridge corresponded to the length of the fibrin scaffold. MSCs that were delivered via intraspinal injection were mainly oriented perpendicular to the plane of the spinal cord and remained largely restricted to the lesion site. Host neurites within the injected MSC graft were also oriented perpendicular to the plane of the spinal cord.

## Highlights

• MSCs can be delivered to injured spinal cord through use of a fibrin scaffold.
• Scaffold-delivered MSCs form longitudinally-aligned layers over the lesion site.
• Regenerating axons enter scaffold-delivered grafts and grow longitudinally.
• MSCs delivered via injection orient perpendicular to the plane of the spinal cord.
• Regenerating axons in injected grafts grow perpendicular to the plane of the cord.

## 1 Introduction

The failure of axons to regrow following spinal cord injury has been attributed to a number of factors; these include the hostile environment, the presence of inhibitory molecules, the absence of a permissive substrate through which regenerating axons can project across the lesion, and insufficient trophic support [1]. Transplantation of bone marrow-derived mesenchymal stromal cells (MSCs) may help to overcome some of these factors. A major advantage of using MSCs over most other cell types is that MSCs can be autologously sourced, rendering them non-immunogenic. MSCs are also easily expanded *ex vivo*, making them attractive for clinical use. MSCs have been shown to have a beneficial effect in a variety of CNS injury models, including traumatic brain injury [2], stroke [3], and spinal cord injury [1,4–10]. The cells are known to release a range of trophic factors, such as BDNF, NGF, VEGF, FGF-2, TGF-β, IGF-1, brain natriuretic peptide, and stromal cell-derived factor-1 [11]; these factors can contribute to neuroprotection, stimulate neovascularisation, and facilitate wound repair. There is also evidence that MSCs induce axonal sprouting and remyelination [12] as well as modulate the immune response [13,14], however, the precise mechanisms by which MSCs promote healing remain unclear.

A number of studies have examined the use of MSCs in acute or chronic injury models of the spinal cord. In both models, MSCs have been shown to promote significant functional recovery [4–6,9]. All of these studies used intraspinal injection as the delivery method for the cells. This type of injection has been shown to be far more effective than intravenous administration for chronic spinal cord injuries [15]. However, intraspinal injection may not be the optimum approach for delivering cells to the spinal cord [16]. Previous research in the eye has shown that using a cell scaffold can improve cell survival and integration after transplantation [17]. A scaffold provides the cells with an adherent substrate and may help to prevent anoikis [18]. The focus of this study was to investigate a novel fibrin scaffold as a new method of delivering cells. MSCs growing as a single layer on top of a fibrin sheet were laid over top of the injured spinal cord.

## 2 Methods and materials

### 2.1 Manufacture of EDC-fibrin scaffold

7.8 mg of Tisseel powder (Baxter Healthcare Ltd.), containing 4.8 mg of human fibrinogen, was dissolved in 27 μL of aprotinin (Baxter Healthcare Ltd.). A solution of EDC (N-(3-dimethylaminopropyl)-N-ethyl-carbodiimide hydrochloride; Sigma–Aldrich) and NHS (N-hydroxysuccinimide; Sigma–Aldrich) was then prepared: 62.5 μL of dH_2_O was added to a mixture of 7.5 mg of NHS and 12.5 mg of EDC. 6 μL of the EDC/NHS solution was then physically mixed into the fibrinogen solution using a micropipette tip for approximately two minutes until the gel began to solidify. Next, the ball of fibrinogen was compressed between two glass plates and allowed to set for 15 min at room temperature. PBS was then added to the fibrin sheet to rehydrate it before moving it to a 50 mL plastic tube containing PBS. The fibrin was soaked in PBS for at least two hours before cutting it into two rectangular sheets (6 mm × 4 mm, henceforth referred to as fibrin scaffolds) using a razor blade under stereomicroscope observation. The fibrin scaffolds were subsequently sterilised overnight at 4 °C in a PBS solution containing 1% chloroform (Fisher Scientific). The scaffolds were then washed repeatedly with large volumes of sterile PBS for at least four days to remove any trace of the chloroform and crosslinking reagents.

### 2.2 MSC culture and plating onto a fibrin scaffold

Rat GFP^+^ bone marrow-derived MSCs were isolated previously [19]. The cells were grown *in vitro* in vented T75 tissue culture flasks (Asahi Glass Co. Ltd). Dulbecco's modified eagle's medium (DMEM, Invitrogen) containing 10% fetal calf serum (Invitrogen), penicillin, streptomycin, and amphotericin B (100 units, 0.1 mg, and 0.25 μg respectively; Sigma–Aldrich) was used as the culture media. Cells were passaged whenever they became 90% confluent. The MSCs were detached from the culture flask using 0.05% trypsin-EDTA in HBSS (without Ca^2+^ or Mg^2+^, Invitrogen). When plating the cells onto a fibrin scaffold, a suspension of MSCs in media was prepared. A sterile fibrin scaffold was pinned to a cell strainer (BD Biosciences) using stainless steel minutien pins (0.1 mm diameter/10 mm length, InterFocus) and submerged in 7.5 mL of culture media contained within one well of a six well plate (Nunc). 700,000 cells were then added to the media on top of the scaffold and the cells were allowed to adhere and proliferate for approximately 24 h in a 37 °C incubator (containing 5% CO_2_).

### 2.3 Lesioning of rat spinal cord and application of MSCs

All procedures were performed in compliance with the UK Animals (Scientific procedures) Act 1986 and institutional guidelines. Female Sprague Dawley rats (200–250 g) were deeply anesthetised with 1–2% isoflurane in a mixture of 25% nitrous oxide and 50% oxygen. A spinal cord injury was performed as previously described [20]. Briefly, a C4 laminectomy was performed to expose the spinal cord. In this case, the laminectomy extended to most of C3 and C5 vertebrae in order to accommodate the scaffold. The dura overlying the spinal cord at the C3, C4, and C5 levels was removed. A cut was made with the tips of sharpened fine forceps (Dumont No. 5) inserted 2 mm in depth into the spinal cord parenchyma spanning the gap between the dorsal root entries. The injury included the descending dorsal corticospinal tracts and the ascending sensory dorsal columns [21]. Four animals received an injection of MSCs, 4 μL of cells (4.3 × 10^6^ cells/mL) was injected directly into the lesion site through a pulled glass capillary needle with a tip diameter of approximately 100 μm (Harvard Instruments) at a rate of 6 μL/h. Three animals received MSCs via fibrin scaffold, the scaffold was unpinned from the cell strainer (pulling the pins out through the bottom of the strainer) and lifted using a paintbrush, then laid over top of the lesion site (cell-side down). Both the scaffold and injection contained approximately the same number of cells. Quantification of the number of cells growing on fibrin scaffolds after 24 h of incubation was done in order to determine the number of cells to inject (data not shown).

### 2.4 Immunohistochemical staining of GFP and GAP43 in lesioned spinal cord treated with GFP^+^ MSCs

Animals were sacrificed three weeks after MSC treatment/injury and transcardially perfused with 4% PFA. The spinal cord from C2-C6 was dissected out of each perfused animal and post-fixed overnight in 4% PFA (Sigma–Aldrich) and then cryoprotected in 30% sucrose (Sigma–Aldrich). The spinal cord tissue was then embedded in O.C.T™ Compound (Sakura Finetek) and cryosectioned longitudinally on a Leica CM 3050S cryostat. 20 μm thick sections were mounted on a series of SuperFrost^®^ glass slides (VWR), such that each slide possessed sections from throughout the spinal cord. Slides were stored at −20 °C. Slides from each animal were stained with either chicken anti-GFP antibody (1:1000 dilution, Millipore) and mouse anti-GAP43 antibody (1:500 dilution, Millipore) or chicken anti-GFP antibody and rabbit anti-neurofilament heavy chain antibody (1:500 dilution, ENZO). Antibody dilutions were made in PBS containing 10% heat-inactivated horse serum (Sigma–Aldrich) and 0.2% Triton X–100 (Sigma–Aldrich). Primary antibodies were labelled with anti-chicken Alexa Fluor 488 antibody (1:1000 dilution, Invitrogen), anti-mouse Alexa Fluor 555 antibody (1:1000 dilution, Invitrogen), and anti-rabbit Alexa Fluor 555 antibody (1:1000 dilution, Invitrogen).

## 3 Results

### 3.1 Delivery of MSCs to spinal cord via fibrin scaffold

**Fig. 1:**
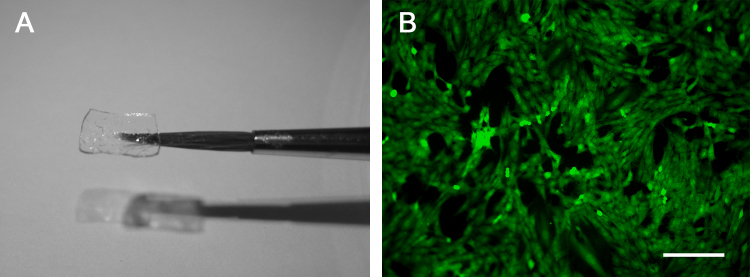
EDC-fibrin scaffold. (A) Suspended in the air with a paintbrush. Scaffold dimensions are 6 mm × 4 mm. (B) GFP+ MSCs growing on the surface of a scaffold *in vitro*. Scale bar represents 200 μm.

**Fig. 2:**
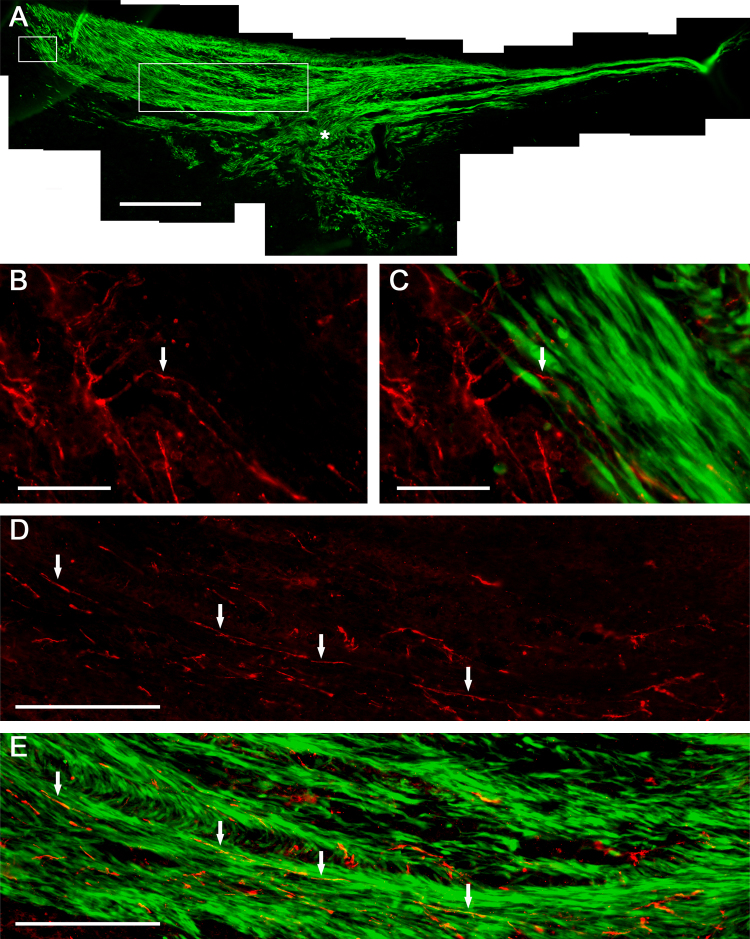
MSC graft delivered to injured spinal cord via fibrin scaffold (3 weeks post-implantation). (A) Entire graft stained with anti-GFP antibody. Asterisk indicates the lesion site. (B and C) Magnified area of graft (designated by small box in A), showing GAP43 positive neurites (red) entering the MSC graft (green). The arrow points to a neurite that changes direction after entering the graft. (D and E) Magnified area of graft (designated by large box in A), shows GAP43 positive neurites growing within the MSC graft. Arrows point toward neurites and serve as reference points. Scale bars represent: 500 μm (A), 50 μm (B and C), 200 μm (D and E).

Thin sheets of EDC-fibrin were created for use as cell scaffolds (Fig. 1A). MSCs were found to adhere to and grow on the surface of the scaffolds *in vitro* (Fig. 1B). The cells were implanted *in vivo* as a single layer, growing on the surface of the fibrin. After implantation, the MSCs proliferated between the fibrin scaffold and the spinal cord to form a multi-layered graft. The cells completely filled the lesion site and grew over top of a significant amount of unlesioned spinal cord tissue flanking the injury (Fig. 2A). The length of the MSC graft (after three weeks *in vivo*) was comparable to the length of the initial fibrin scaffold (5.82 ± 0.92 mm versus 6 mm; mean ± SD, *N* = 3). The scaffold remained intact *in vivo* for at least three weeks. The three animals that received MSCs via scaffold and four animals that received MSCs via injection were assessed qualitatively for MSC orientation and neurite outgrowth. At the boundary between the MSC graft and the host spinal cord, GAP43^+^ neurites were found to migrate into the MSC graft and change their direction of growth, conforming to the architecture of the graft; some neurites were seen to enter the graft at a significant distance from the lesion site (Fig. 2B and C). The graft was mainly composed of longitudinally-aligned layers of MSCs, neurites found within the graft mostly conformed to this architecture (Fig. 2D and E). The MSCs that filled in the lesion site did show less organized longitudinal architecture than the rest of the graft; as expected the axons that entered this region of the graft also showed more variability in their direction of growth.

### 3.2 Delivery of MSCs to spinal cord via intraspinal injection

**Fig. 3:**
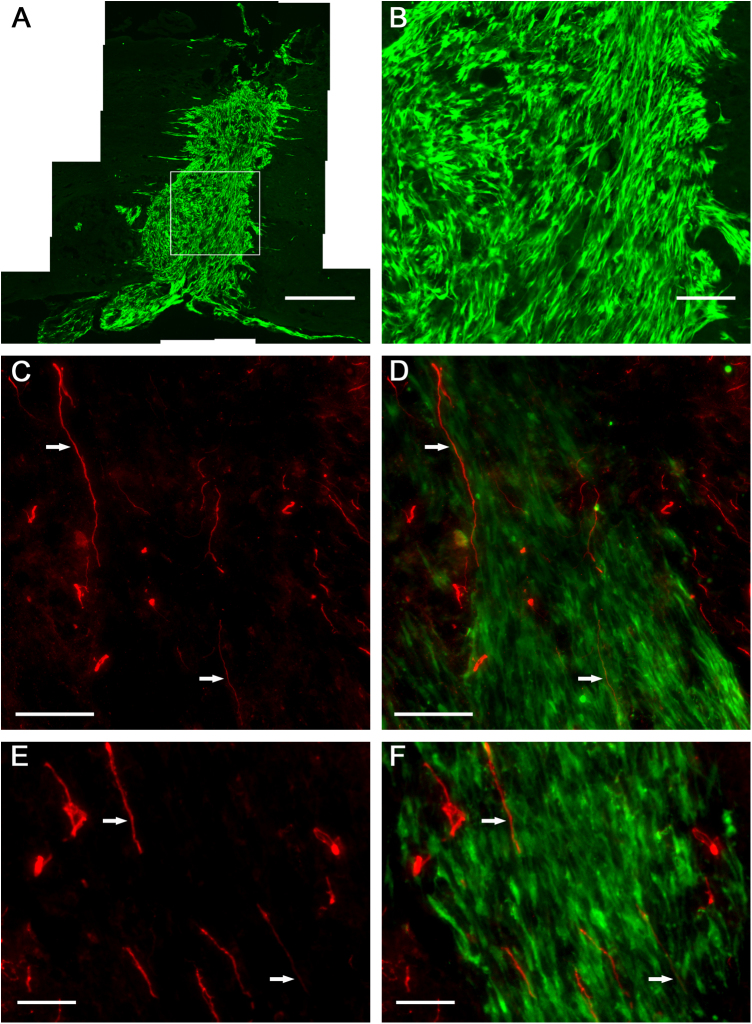
MSC graft delivered to injured spinal cord via intraspinal injection (3 weeks post-injection). (A) An entire graft stained with anti-GFP antibody. (B) Magnified area of graft (designated by box in A). Neurites stained for neurofilament (C and D) and GAP43 (E and F) growing within a graft. Arrows point toward neurites and serve as reference points. Scale bars represent: 500 μm (A), 100 μm (B–D), 50 μm (E and F).

MSCs injected into the spinal cord remained viable and integrated into the tissue, completely filling the lesion site. After three weeks *in vivo*, the cells were found to be largely restricted to the injury site (Fig. 3A). The longitudinal length of an injected graft was 1.54 ± 1.33 mm (mean ± SD, *N* = 4). This is significantly less than the length of grafts formed when a fibrin scaffold was used to deliver the MSCs (*p* < 0.005; unpaired, two-tailed *T*-Test). Many of the MSCs delivered via injection adopted an orientation that was perpendicular to the plane of the spinal cord, many cells also assumed random orientations (Fig. 3B). Host neurites were found to migrate into the MSC graft (Fig. 3C–F). Many neurofilament^+^ neurites (Fig. 3C and D) and GAP43^+^ neurites (Fig. 3E and F) found within an injected graft were seen to grow perpendicular to the plane of the spinal cord, in accordance with the architecture of the MSCs.

## 4 Discussion

In animal models of spinal cord injury, intraspinal injection is a common method of delivering therapeutic cells. It is simple and often effective at engrafting cells. However, there are some concerns that this method may cause additional damage to the spinal cord [16]. The purpose of this study was to determine if a biodegradable fibrin scaffold could be used instead of direct injection to deliver MSCs.

A novel formulation of fibrin, crosslinked with EDC, was developed for use as the scaffolding material. Fibrin is a clinically approved material that is routinely used in human patients. EDC is a zero-length crosslinker that has been used extensively with collagen [22–24]. Recently, artificial corneas made from EDC-crosslinked collagen entered human clinical trials [25]. We found that it was possible to create thin sheets of EDC-crosslinked fibrin, which supported the attachment and proliferation of MSCs on their surface. The scaffolds were rigid enough to maintain their shape while handling and flexible enough to conform to the spinal cord upon implantation. Scaffolds were found to slowly degrade *in vivo*, lasting approximately six weeks.

MSCs delivered via scaffold formed stratified layers that bridged the lesion site. The cells had no preferred orientation prior to implantation. It is possible that the cells re-orient after implantation into a more organized longitudinal direction. Schwann cell studies utilizing matrices have previously shown that these cells will orient longitudinally relative to the spinal cord [26,27]. Furthermore, Hofstetter et al. showed that MSCs can form parallel cords that orient longitudinally in the spinal cord [7]. Another possibility is that the MSCs remain randomly oriented, and as they proliferate from a single layer *in vitro* into a multi-layered graft *in vivo*, they create longitudinally-aligned layers of cells. If we were to view the MSCs within each layer from a top-down view, we may find that the cells look disorganized, similar to that seen *in vitro* (Fig. 1B). We can only conclude from our data that the layers of cells are longitudinally oriented.

The length of the MSC graft corresponded to the length of the fibrin scaffold; therefore, it may be possible to control how far the cellular bridge extends past the edges of the lesion site by simply changing the length of the scaffold. In the present study, a significant amount of unlesioned spinal cord tissue, flanking the injury site, was present beneath the MSC graft. Host neurites within this unlesioned tissue were found to migrate into the graft. In addition, the layered architecture of the MSCs appeared to promote longitudinal growth of neurites within the graft. Further investigation will be needed to determine whether these factors (i.e. graft contact with unlesioned tissue and the layered architecture of the MSCs) facilitate regeneration of damaged spinal cord.

In contrast to MSCs delivered by the scaffold, MSCs delivered via injection largely remained restricted to the lesion site and were not seen to contact significant amounts of unlesioned spinal cord tissue. The cellular bridges formed by MSCs delivered by the scaffolds were nearly four times longer than the grafts formed by injected cells. Furthermore, the orientation of the engrafted cells differed significantly between the two groups. Whereas MSCs delivered via scaffold formed longitudinally-aligned layers, the MSCs delivered via injection often oriented perpendicular to the plane of the spinal cord. Neurites found within the injected cell graft also grew perpendicular to the plane of the spinal cord. Previous research has shown that regenerating neurites will enter MSC grafts within the spinal cord and use the orientation of the MSCs as a guide to determine the direction of growth [7]. Furthermore, the chaotic architecture of cells often seen within a spinal cord lesion site promotes haphazard orientations of regenerating axons within the lesion; disoriented axons are unlikely to facilitate much recovery [16,28].

It remains to be seen whether the differences between the two types of MSC graft (i.e. the injected graft and the graft delivered by the scaffold) affect the ability of the spinal cord to regenerate following injury. It would also be useful to evaluate the fibrin system using a contusion injury model instead of the vertical cut model used in this study. It is possible that the cut injury model resulted in greater infiltration of fibroblasts and the formation of a vertical scar, which could have influenced the orientation of the MSCs. We consider the fibrin delivery system promising in many regards, particularly with respect to MSC orientation and neurite outgrowth, but it will require further studies to validate any advantages over existing delivery methods.
